# Non-coding RNA derived from a conservative subtelomeric tandem repeat in chicken and Japanese quail somatic cells

**DOI:** 10.1186/s13039-014-0102-7

**Published:** 2014-12-23

**Authors:** Irina Trofimova, Darya Popova, Elena Vasilevskaya, Alla Krasikova

**Affiliations:** Department of Cytology and Histology, Saint-Petersburg State University, Oranienbaumskoie sch. 2, Stary Peterhof, 198504 Saint-Petersburg, Russia

**Keywords:** Cell cycle, Cell nucleus, Chicken, Mitosis, Non-coding RNA, Subtelomere, Tandem repeats, Transcription

## Abstract

**Background:**

Subtelomeres are located close to the ends of chromosomes and organized by tandemly repetitive sequences, duplicated copies of genes, pseudogenes and retrotransposons. Transcriptional activity of tandemly organized DNA at terminal chromosomal regions and the distribution of subtelomere-derived non-coding RNAs are poorly investigated. Here we aimed to analyze transcriptional activity of subtelomeric tandem repeat in somatic tissues and cultured cells of birds. We focused on tissue-specific differences of subtelomeric repeats transcription, structure of the resulting transcripts and the behavior of subtelomere-derived RNA during mitosis.

**Results:**

Transcriptional activity of short subtelomeric PO41 (“pattern of 41 bp”) tandem repeat in the somatic and cultured cells of chicken (*Gallus gallus domesticus*) and Japanese quail (*Coturnix coturnix japonica*) was examined using RNA fluorescence *in situ* hybridization approach. We discovered transcripts from both strands of the PO41 repeat in chicken MDCC-MSB1 cells as well as in chicken and Japanese quail somatic tissues, such as tissues of cerebellum, telencephalon, muscles, oviduct, small and large intestine. Normal somatic and transformed cells demonstrate similar distribution of PO41 repeat transcripts in interphase nuclei. We revealed one or two major foci of PO41 repeat transcripts associated with RNA polymerase II, representing nascent RNA, and dispersed PO41 repeat transcripts localized in euchromatin or interchromatin space, representing released RNA. During mitosis PO41 non-coding RNA distribute between condensed chromosomes till anaphase, when they concentrate at the cleavage plane. At telophase, clusters of PO41 RNA surround terminal regions of chromosomes. Treatments with RNases of different substrate specificity indicate that PO41 repeat transcripts are single-stranded RNAs with short double-stranded regions due to appearance of inverted repeats.

**Conclusion:**

Transcription of a subtelomeric tandem repeat in avian somatic cells is reported here for the first time. PO41 repeat transcription is conserved among Galliformes and has similar pattern in somatic tissues. We demonstrated redistribution of non-coding PO41 RNA occurring during the cell cycle. Potential regulatory role of the PO41 repeat transcripts in RNA-dependent process of subtelomere heterochromatin maintenance is discussed.

**Electronic supplementary material:**

The online version of this article (doi:10.1186/s13039-014-0102-7) contains supplementary material, which is available to authorized users.

## Background

Satellite DNA consists of non-protein-coding tandemly repetitive sequences and represents an abundant DNA fraction of eukaryotic genomes. For a long time transcriptional silencing was considered as a fundamental property of satellite DNA, and occasional reports on satellite DNA transcription remained unnoticed [1–7]. Now it is obvious that transcription of tandemly repeated DNA takes place in many organisms. Furthermore satellite DNA transcription is tissue- and cell type-specific, depends on stages of cell cycle, cell differentiation and ontogenesis, and can be stress-induced [8–16]. RNA-transcripts from tandemly repeated DNA play a significant role in heterochromatin establishment and maintenance, chromocenter formation, gene regulation, centromere specification, telomere functioning, cell fate determination, stress response, and specific RNA cleavage [17–19].

However, despite great interest to the problem of satellite DNA transcription, distribution and functions of RNA-transcripts complementary to subtelomeric regions of chromosomes are poorly investigated. Subtelomers are located close to the ends of chromosomes and organized by tandemly repetitive sequences, duplicated copies of genes, pseudogenes and retrotransposons [20,21]. Subtelomeres participate in DNA repair and recombination, realization of antigenic variation, maintenance of chromosome ends stability even in the absence of telomeric DNA sequences or telomerase activity [20,21].

Previous studies have demonstrated transcription of repetitive sequences located in subtelomere regions of chromosomes, for instance transcription of subtelomeric repeats in *Plasmodium falciparum* [22] and fission yeast [23], and transcription of tandem subtelomeric repeats in HeLa cells [24], *Leishmania infantum* [25] and budding yeast [26]. Transcripts from subtelomeric repeats were suggested to be involved in heterochromatin formation in fission yeast [23] and cell differentiation in malarial plasmodium *P. falciparum* [22]. Besides, transcripts from tandem subtelomeric repeats probably participate in regulation of gene expression in *L. infantum* [25]. However, biological role of non-coding RNA (ncRNA) derived from subtelomeric repeats in Vertebrata remains largely unknown.

To address this problem we analyzed transcriptional activity of subtelomeric tandem repeat in birds. In chicken genome, several types of subtelomeric tandem repeats are characterized in detail [27]. Moreover, a chromosome-wide distributed transcription of chicken subtelomeric Z-Macro-satellite as well as chicken and Japanese quail PO41 (‘pattern of 41 bp’) tandem repeat was demonstrated in growing oocytes [10,28]. Using RNA fluorescent *in situ* hybridization (FISH) authors revealed transcripts of the subtelomeric repeats in subterminal transcription units on giant lampbrush chromosomes isolated from growing chicken and Japanese quail oocytes. The high rate of PO41 repeat transcription was confirmed by BrUTP incorporation [10]. Furthermore, since transcripts from both strands of PO41 repeat appear on lampbrush lateral loops, it was speculated that resulting long double-stranded RNA might be involved into small interfering RNA (siRNA) processing pathway [10].

Here we aimed to characterize transcripts of subtelomeric tandem PO41 repeat in normal somatic cells of domestic chicken and Japanese quail and chicken transformed cell line. We focused on three questions: does transcription of PO41 repeat occur in somatic cells of chicken and Japanese quail, whether transcription of PO41 repeat has tissue-specific differences, and whether transcription of PO41 repeat differs between these two representatives of Galliformes. Furthermore we investigated the distribution of subtelomere repeat transcripts at different stages of cell cycle, relative localization of subtelomere RNA and known nuclear domains and analyzed structure of the transcripts using treatments with RNases of different substrate specificity. Subtelomeric tandem PO41 repeat was chosen due to its high DNA sequence conservation and similar chromosomal distribution in at least three species of the order Galliformes, which suggests that there could be important invariable functions of PO41 repeat transcripts. We have discovered transcripts of both strands of subtelomeric PO41 tandem repeat in different chicken and Japanese quail cell types. Our results represent the first example of transcription of tandemly highly repetitive DNA sequences in normal somatic and transformed cells in the representatives of birds. Potential structure and regulatory role of subtelomeric PO41 repeat transcripts in heterochromatin establishment and maintenance are discussed.

## Results

### Subtelomeric PO41 repeat is transcribed in chicken lymphoblastoid MDCC-MSB1 cells

**Figure 1:**
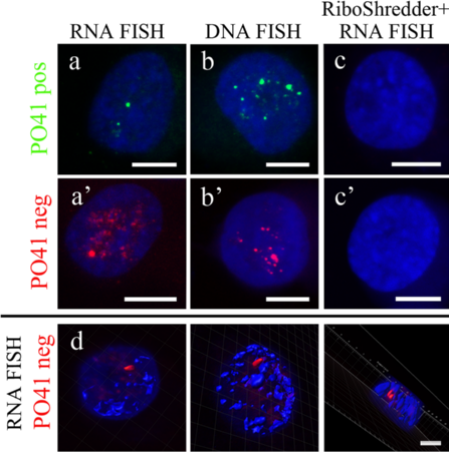
**PO41 tandem repeat is transcribed in chicken lymphoblastoid MDCC-MSB1 cells.** FISH with PO41pos (*green*) and PO41neg (*red*) probes on chicken MDCC-MSB1 cells. **(a, a’)** DNA/RNA hybridization revealed transcripts from both strands of PO41 repeat in interphase nuclei. **(b, b’)** DNA/DNA hybridization (positive control) revealed clusters of PO41 repeat in interphase nuclei. **(c, c’)** RiboShredder RNase cocktail treatment before DNA/RNA hybridization (negative control) removed all hybridization signals. **(d)** 3D reconstruction of interphase nucleus with isosurfaces around G-rich transcripts of PO41 repeat (*red*) and chromatin (*blue*). DNA was counterstained with DAPI. Scale bars: 5 μm **(a-c, a’-c’)**; 3 μm **(d)**.

To study the transcriptional activity of subtelomeric tandem PO41 repeat, we performed FISH according to DNA/RNA hybridization protocol with single stranded oligonucleotide probes (PO41pos and PO41neg) to each strand of the repeat. RNA FISH was used as a reliable approach to reveal specific transcripts in either cultured cells or tissues [29]. At first, we analyzed the transcription of PO41 repeat in chicken MDCC-MSB1 cell line as in one of the few widespread permanent cell lines of birds with a high level of proliferative activity [30]. In MDCC-MSB1 cells, we detected transcripts from both strands of PO41 repeat in interphase nuclei, but not cytoplasm (Figure 1a, a’). The pattern of intranuclear distribution was identical for probes to each of the strands of tandem repeat: transcripts localized predominantly in one, rarely two foci typically observed in euchromatin (~0.59 μm and 0.54 μm for C- and G-rich transcripts respectively) (Figure 1d). It indicates that transcription of PO41 repeat is probably initiated at one locus in a genome. The transcription from multiple sites could not be excluded but remained undetected due to bright signals from the major foci. Since bright foci bearing PO41 repeat transcripts are present in nuclei of all cells, we suggest that PO41 repeat transcription begins in early G_1_ phase. Treatment of MDCC-MSB1 cells with a cocktail of RNases (RiboShredder RNase Blend) before DNA/RNA FISH effectively eliminated all fluorescent signals (Figure 1c, c’) which confirms probes hybridization to RNA-transcripts.

DNA/DNA FISH with the same probes, as expected, detected several PO41 repeat clusters preferentially located close to chromocenters in the internal part of the nucleus (Figure 1b, b’). Indeed, subtelomeric regions of many chromosomes could aggregate in chicken interphase nuclei (A.V.Krasikova, A.V.Maslova, unpublished observations). To avoid detection of signals from remaining transcripts, all images after DNA/DNA FISH were obtained with lower value of the PMT voltage (“Gain” parameter) in comparison with DNA/RNA FISH. Moreover, detection of PO41 RNA in MDCC-MSB1 cells is specific, since dual color RNA FISH with PO41neg and U7 snRNA probes demonstrated that U7 snRNA, which accumulates in histone locus bodies in MDCC-MSB1 cells [31], localized in one or two distinct foci that do not overlap with the major focus of PO41 repeat transcripts (Additional file 1: Figure S2). Thus both C- and G-rich transcripts of subtelomeric 41 bp repeat were revealed in interphase nuclei of chicken lymphoblastoid MDCC-MSB1 cells.

### Nuclear PO41 RNA foci do not correspond to Cajal bodies, splicing speckles and hnRNP K-domains

Since PO41 repeat transcripts form predominantly one major focus in interphase nuclei, we checked whether intranuclear domain enriched with PO41 ncRNA corresponds to well-characterized nuclear structures such as “transcription factories”, clusters of interchromatin granules, nuclear stress bodies (nSBs) [32,33] or Cajal bodies (CBs) [34]. FISH after immunofluorescent staining (immunoFISH) has not revealed any specific accumulation of small nuclear RNAs (snRNA), detected with antibodies against 2,2,7-trimethylguanylated cap (TMG-cap), in the foci concentrating PO41 repeat transcripts (Additional file 1: Figure S1a- a”).

Using immunoFISH we further compared the distribution of PO41 repeat transcripts with the distribution of protein K of heterogeneous nuclear RNP (hnRNP K) which is a marker component of nSBs [32,33]. In MDCC-MSB1 cell nuclei, hnRNP K revealed by 3C2 antibodies forms multiple domains localized in nucleoplasm without overlapping with foci of a high local concentration of PO41 repeat transcripts (Additional file 1: Figure S1b- b”). In MDCC-MSB1 cells, coilin, which is known to be one of the main components of CBs [34], was localized in one or two distinct bodies in each interphase nuclei; brightly labeled PO41 RNA foci and CBs appear in the different parts of the nucleus (Additional file 1: Figure S2).

ImmunoFISH with H14 antibodies against the phosphorylated C-terminal domain of RNA polymerase II produced punctuate pattern in interphase nuclei of MDCC-MSB1 cells (Additional file 1: Figure S1c-c”). Focus enriched with PO41 repeat transcripts was often localized adjacent to small-grained granules containing elongating form of RNA polymerase II. These observations may suggest that in interphase nuclei the PO41 repeat is transcribed by means of RNA polymerase II.

### Redistribution of PO41 repeat transcripts during the cell cycle progression

**Figure 2:**
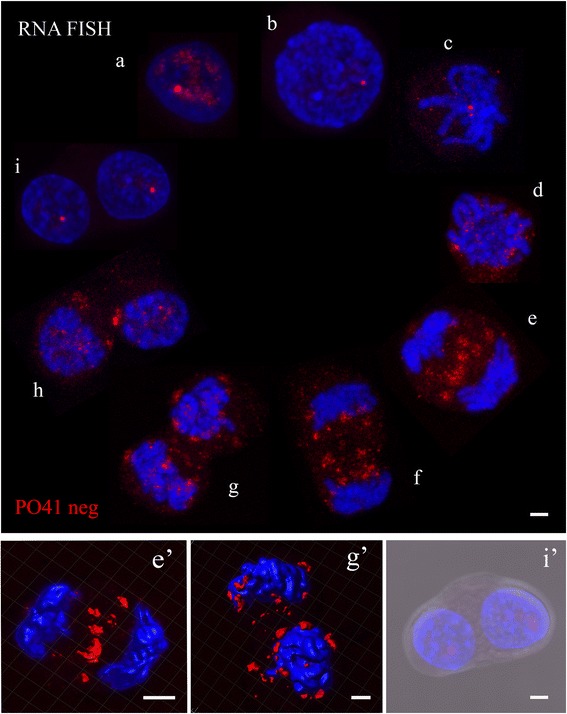
**Redistribution of PO41 RNA during the cell cycle.** DNA/RNA FISH with the PO41neg probe (*red*) representing the redistribution of PO41 RNA during the cell division. MDCC-MSB1 cells at interphase **(a)**, prophase **(b)**, metaphase **(c, d)**, anaphase **(e, f)**, telophase **(g, h)** and cytokinesis **(i, i’)** are shown. Images of anaphase and telophase cells were acquired with higher gain values. 3D reconstructions of MDCC-MSB1 cells at anaphase **(e’)** and telophase **(g’)** stages with isosurfaces around G-rich transcripts of PO41 repeat (*red*) and chromatin (*blue*). DNA was counterstained with DAPI. Scale bars: 3 μm **(a-i, e’)**; 2 μm **(g’)**.

We further examined redistribution of PO41 repeat transcripts during the cell cycle progression in MDCC-MSB1 cells (Figure 2a-i’). For each stage of mitosis (from prophase to telophase) no less than 25 cells were analyzed; at interphase stage more than 100 nuclei were analyzed. During prophase, foci containing transcripts of PO41 repeat were located around condensing chromatin (Figure 2b). At metaphase stage dispersed transcripts of PO41 repeat and one or several more bright foci were distributed between condensed chromosomes (Figure 2c, d). Starting from early anaphase, PO41 repeat transcripts formed compact clusters that were localized in the equatorial zone of the dividing cell without association with separating chromosomes (Figure 2e, e’). At late anaphase, PO41 repeat transcripts started to separate to the poles, while at telophase, transcripts formed clusters, adjacent to but not overlapping with terminal regions of chromosomes (Figure 2f- h, g’). At a cytokinesis stage PO41 RNAs formed local foci in both daughter cells nuclei (Figure 2i, i’) indicating start of transcription. This distribution was similar for both C- and G-rich PO41 repeat transcripts. In summary, we found that PO41 RNA specifically redistributes during the cell cycle progression.

### Sensitivity of PO41 repeat transcripts to RNases with different substrate specificity

**Figure 3:**
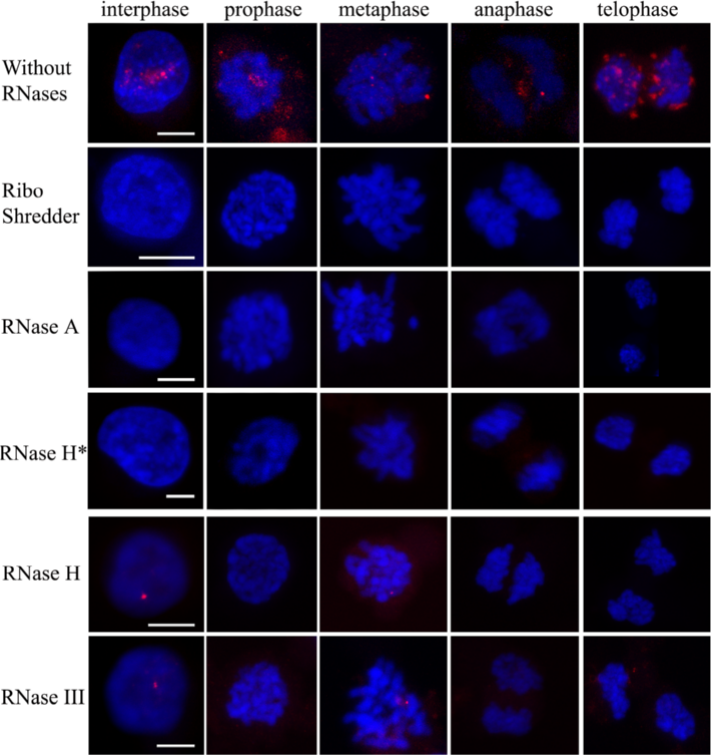
**Sensitivity of G-rich PO41 transcripts during the cell cycle to RNases with different substrate specificity.** Treatment of MDCC-MSB1 cells with different RNases before or after (RNase H*) DNA/RNA FISH with PO41neg (*red*) probes. MDCC-MSB1 cells are shown at interphase, prophase, metaphase, anaphase and telophase stages. RNases used for treatments are indicated on the left. PO41 RNA was degraded by RiboShredder RNases cocktail, RNase A treatments and RNase H treatment performed after FISH. RNase H and RNase III treatments completely removed only dispersed G-rich transcripts at interphase and decreased the intensity of RNA FISH signals at other stages of cell cycle. DNA was counterstained with DAPI. Scale bar: 5 μm.

To characterize the structure of PO41 repeat transcripts, MDCC-MSB1 cells were treated by different RNases before and in the case of RNase H after hybridization (Figure 3 and Additional file 2: Table S1). It is known that RNase A cleaves single-stranded RNAs [35], while RNase H degrades only the RNA strand from RNA/DNA hybrids [36] and RNase III cleaves double-stranded RNA [37]. In interphase nuclei, treatment with RNase A before FISH and treatment with RNase H after FISH completely eliminated all hybridization signals from each probe to PO41 RNA. Treatments with RNase III or RNase H before FISH only decreased the size and fluorescence intensity of PO41 repeat transcripts foci (Figure 3). At the same time, these two RNases eliminated dispersed RNA-transcripts signals.

In dividing MDCC-MSB1 cells, RNase H treatment performed after FISH, RNase A and RiboShredder RNase blend (cocktail of RNases) treatments before FISH removed both C- and G-rich PO41 repeat transcripts at all cell cycle stages. After RNase H or RNase III treatments performed before FISH week signals from hybridization to C- or G-rich transcripts were detected in some metaphase cells (Figure 3; Additional file 2: Table S2). Differential sensitivity of dispersed and concentrated into foci PO41 repeat transcripts to RNases with different substrate specificity may reflect different secondary structure of the transcripts.

### Subtelomeric PO41 repeat is transcribed in chicken somatic tissues

To compare PO41 repeat transcription between transformed and normal somatic cells and to analyze the distribution of the resulting transcripts in different cell types within one tissue, we performed RNA FISH on histological cryosections and 3D-preserved fragments of skeletal muscles, oviduct, brain (telencephalon, cerebellum), small and large intestine.

**Figure 4:**
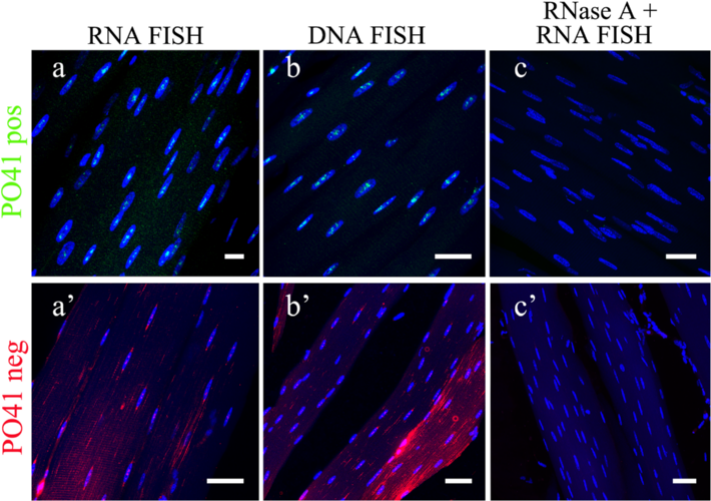
**PO41 repeat is transcribed in chicken skeletal muscle cells.** FISH with PO41pos (*green*, upper row) and PO41neg (*red*, bottom row) probes on skeletal muscle cryosections. **(a, a’)** DNA/RNA hybridization revealed transcripts from both strands of PO41 repeat in cell nuclei. **(b, b’)** DNA/DNA hybridization (positive control) revealed clusters of PO41 repeat in all cell nuclei. **(c, c’)** RNase A treatment before DNA/RNA hybridization (negative control) removed all hybridization signals. Nuclei were counterstained with DAPI. Scale bars: 10 μm **(a)**; 20 μm **(b, c, a’)**; 30 μm **(b’, c’)**.

**Figure 5:**
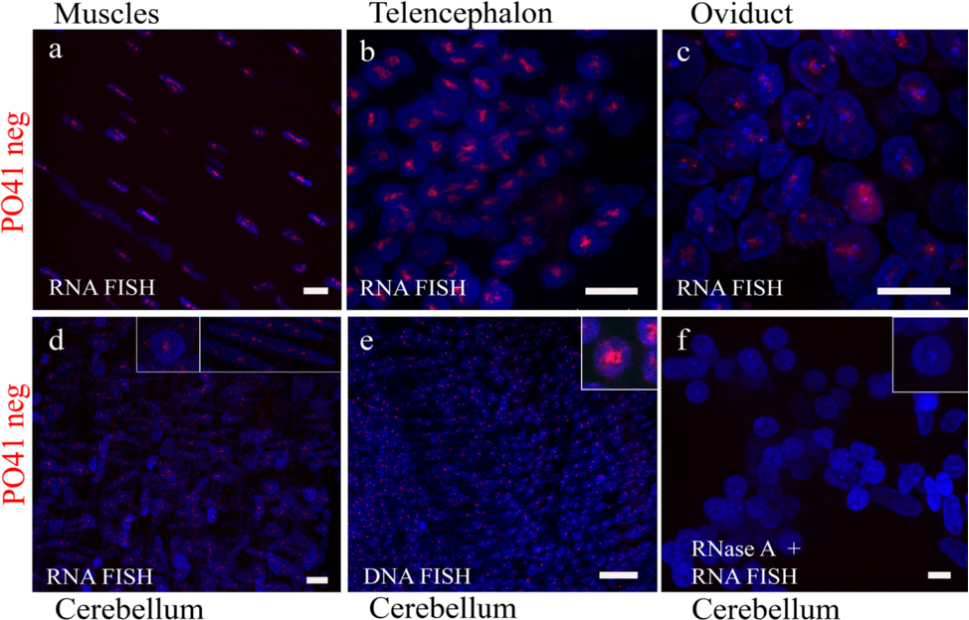
**PO41 repeat is transcribed in chicken somatic tissues.** **(a-d)** 3D DNA/RNA FISH with PO41neg probe (*red*) on whole mount fragments of chicken somatic tissues revealed PO41 repeat transcripts in cell nuclei of muscles **(a)**, brain **(b, d)** and oviduct **(c)**.** (e)** 3D DNA/DNA FISH (positive control) and **(f)** 3D DNA/RNA FISH after RNase A treatment (negative control) are shown for fragments of cerebellum. Nuclei were counterstained with DAPI. Scale bars: 10 μm **(a, b, d, f)**, 20 μm **(e, c)**.

Using DNA/RNA FISH we detected both C- and G-rich transcripts of PO41 repeat within cell nuclei of somatic tissues (Additional file 2: Table S3). In skeletal muscles, both on cryosections and in whole mount tissue fragments, transcripts from both strands formed predominantly from one to three nuclear foci localized in euchromatin (Figure 4a, a’; Figure 5a; Additional file 3: Video 1). In oviduct, the distribution of PO41 RNA was similar: in all cells of oviduct, C- and G-rich transcripts of PO41 repeat formed one or two nuclear foci (Figure 5c; Additional file 1: Figure S3a, a’). All signals were sensitive to RNase A treatment (Figure 4c, c’; Additional file 1: Figure S3c, c’) and were absent at autofluorescence controls. For both probes, FISH according to DNA/DNA hybridization protocol showed several clusters of PO41 repeat in the nuclei of all cell types in skeletal muscles and oviduct (Figure 4b, b’; Additional file 1: Figure S3b, b’). Thus, we conclude that C- and G-rich transcripts complementary to PO41 repeat appear in chicken skeletal muscles and oviduct.

We further examined PO41 repeat transcription in brain tissues. In the cell nuclei of all layers of cerebellum (Figure 5d; Additional file 1: Figure S4a, a’; Additional file 4: Video 2) and telencephalon (Figure 5b; Additional file 1: Figure S5a, a’), both on cryosections and in whole mount tissue fragments, one or two foci of C- and G-rich PO41 repeat transcripts were detected. Inside cell nuclei, foci concentrating PO41 RNA were located in euchromatin or in the nucleoplasm, and in case of telencephalon – close to large central chromocenter. Small cytoplasmic foci were detected around large nuclei of Purkinje neurons in cerebellum. Notably cytoplasmic signals around Purkinje neurons nuclei were RNase A stable (Additional file 1: Figure S4c, c’) and were detected at autofluorescent controls (tissue sections which were subjected to all treatments except hybridization with the probes and post-hybridization washes; Additional file 1: Figure S4d, d’). According to these data we conclude that fluorescence of cytoplasmic granules in Purkinje neurons is not a result of hybridization with PO41 probes.

In contrast, the intranuclear signals resulted from specific probe hybridization to nucleic acids (confirmed by DNA/DNA hybridization) (Figure 5e; Additional file 1: Figure S4b, b’, S5b, b’), and were RNase A sensitive in all cellular layers of the cerebellum and telencephalon (Figure 5f; Additional file 1: Figure S4c, S5c). The only exception were RNase A stable signals in telencephalon and cerebellum cryosections (but not whole mount preparations) in case of hybridization with the PO41neg probe (Figure S4c’, Additional file 1: Figure S5c’). Moreover, on telencephalon cryosections, signals from both probes could be detected after RNase H or RNase III treatment (Additional file 1: Figure S6b, b’, c, c’). RiboShredder RNase blend digested only RNA hybridizing with PO41pos probe, but not with PO41neg probe (Additional file 1: Figure S6a, a’). Signals were detected regardless of when treatments with RNase A or RiboShredder RNase blend were made – before or after fixation of the tissues. We speculate that preservation of the G-rich transcripts in telencephalon cryosections after RNases treatments resulted not only from different sample preparation protocols, but also from secondary structure of transcripts inaccessible for the enzymes. Autofluorescence of vessels on cryosections was observed in channels for both fluorochromes used. These observations suggest the presence of PO41 repeat transcription in chicken brain tissues.

**Figure 6:**
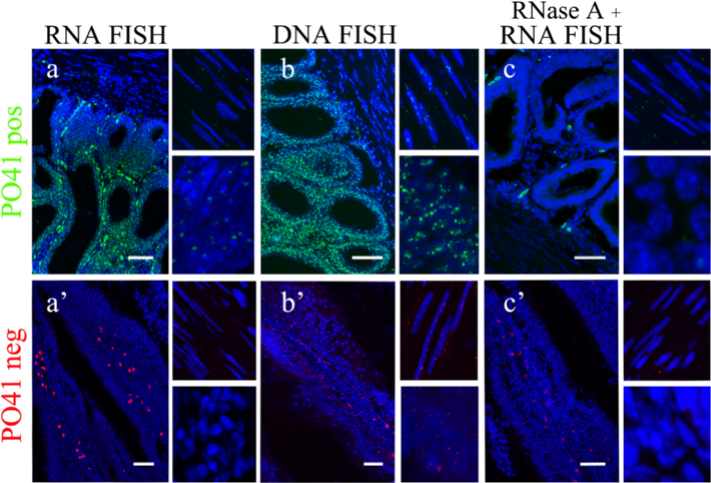
**C-rich transcripts of PO41 repeat in chicken small intestine cells.** FISH with PO41pos (*green*, upper row) and PO41neg (*red*, bottom row) probes on small intestine cryosections. **(a, a’)** DNA/RNA hybridization revealed C-rich (*green*), but not G-rich (*red*) PO41 repeat transcripts in mucous membrane of chicken small intestine. **(b, b’)** DNA/DNA hybridization (positive control) revealed clusters of PO41 repeat in all cell nuclei. **(c, c’)** RNase A treatment before DNA/RNA hybridization (negative control) removed hybridization signals with PO41pos probe from the cell nuclei, but didn’t remove signals in cytoplasmic granules within the mucous membrane. Right-hand images demonstrate enlarged fragments of muscles layer (upper panels) and mucous membrane (bottom panels) of small intestine. Nuclei were counterstained with DAPI. Scale bar: 40 μm.

In a small and large intestine of chicken, RNase A sensitive C-rich but not G-rich transcripts of PO41 repeat were found in the cell nuclei (Figure 6a, c). In all samples of small and large intestine, bright cytoplasmatic granules in mucous membrane were also observed (Figure 6a’). However, the cytoplasmatic granules were observed in positive (DNA/DNA hybridization) (Figure 6b’) and negative (RNase A treatment) (Figure 6c’) controls. In the cells of circular layer of muscles membrane we detected other type of cytoplasmic granules located close to the nuclei (Figure 6). RNase H or RNase III treatments performed before hybridization did not remove nuclear C-rich PO41 repeat transcripts and cytoplasmatic granules on cryosections from small intestine (Additional file 1: Figure S7b, b’, s7c, c’), while RiboShredder RNase cocktail digested C-rich PO41 repeat transcripts (Additional file 1: Figure S7a, a’). At autofluorescent control preparations, cytoplasmic granules were also detected in muscles membrane, in crypt and villi of the mucous membrane, but did not form distinct structures (Additional file 1: Figure S7d, d’). Eventually, in small and large intestine only C-rich transcripts of PO41 repeat were revealed.

Based on these collective results we conclude that C-rich RNAs complementary to PO41 repeat are synthesized in chicken somatic tissues: skeletal muscle, oviduct, cerebellum, telencephalon, small and large intestine. G-rich PO41 repeat transcripts are being formed in all tissues under investigation except small and large intestine.

### Subtelomeric PO41 repeat is transcribed in Japanese quail somatic tissues

**Figure 7:**
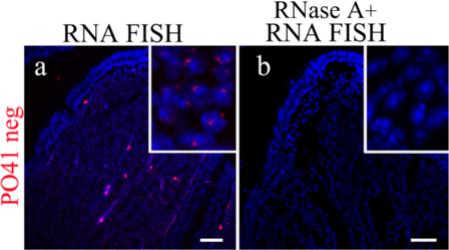
**PO41 repeat is transcribed in Japanese quail somatic cells.** FISH with PO41neg (*red*) probe on Japanese quail oviduct cryosections (bottom row). **(a)** DNA/RNA hybridization revealed transcripts of PO41 repeat in all cell layers. **(b)** RNase A treatment before DNA/RNA hybridization (negative control) removed nuclear hybridization signals. Nuclei were counterstained with DAPI. Scale bars: 40 μm.

Since PO41 repeat is conserved between chicken and Japanese quail and localizes at the homologous chromosomal regions in these species [10] we performed comparative analysis of its transcriptional activity in Japanese quail somatic tissues. FISH with the PO41 oligonucleotide probes was performed on whole mount tissue fragment preparations and cryosections of skeletal muscles, oviduct, brain (cerebellum and telencephalon), small and large intestine from quail females. Using confocal laser scanning microscopy we found C-rich transcripts of the PO41 repeat in all studied tissues and G-rich transcripts in all tissues except small and large intestine. Important to note, that the pattern of transcripts distribution in cell layers of tissues was similar to that in chicken. In cell nuclei of skeletal muscles, brain, oviduct, small and large intestine one or several major foci of PO41 repeat transcripts were located in euchromatin. RNase A pretreatment completely eliminated hybridization of PO41pos and PO41neg probes with nuclear transcripts in all tissues (Figure 7) with one exception: failure of elimination of PO41neg probe was observed in brain tissues. Thus PO41 repeat demonstrate the same tissue-specific pattern of transcription in chicken and Japanese quail.

## Discussion

Here we present new data on the transcription of tandemly organized subtelomeric DNA. We revealed transcripts from both strands of subtelomeric PO41 tandem repeat in normal somatic and transformed cells of the species of the order Galliformes and demonstrated redistribution of non-coding PO41 RNA occurring during the cell cycle.

### Tissue-specific pattern of PO41 repeat transcription is identical in two representatives of the order Galliformes

Transcription of PO41 repeat in somatic cells has not yet been investigated. Previous study has demonstrated that PO41 repeat is intensively transcribed from multiple loci at lampbrush chromosomes in growing oocytes of both chicken and Japanese quail [10]. The distribution and DNA sequence of PO41 repeat in *G. g. domesticus* and *C. c. japonica* genomes are very similar [10,27]. The alteration is in additional sites of PO41 repeat arrays in pericentromeric regions of Japanese quail microchromosomes [10]. We expected that the profile of PO41 repeat transcription would be similar between chicken and Japanese quail somatic tissues. Indeed, we proved that transcription of both strands of PO41 repeat occurs both in chicken and Japanese quail tissues under investigation, except small and large intestine where only C-rich transcripts were detected. Moreover, in both species transcription has similar cell-type specificity. PO41 RNA is abundant in the same cell types of all studied tissues (cerebellum, telencephalon, skeletal muscles, oviduct, small and large intestine), suggesting common functions of PO41 RNAs.

### PO41 RNA is predominantly single-stranded and can form hairpin structures

**Figure 8:**
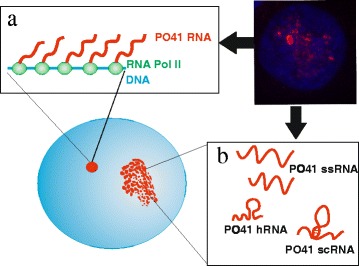
**Hypothetical scheme of PO41 repeat transcripts structure in different nuclear compartments.** PO41 repeat transcripts are shown in *red*, elongating form of RNA polymerase II – in *green*, nucleoplasm and chromatin – in *blue*. Major foci of PO41 repeat transcripts associated with RNA polymerase II presumably represent nascent RNA **(a)** while dispersed PO41 repeat transcripts localized in euchromatin or interchromatin space presumably represent released RNA **(b)**. In interphase nucleus, PO41 RNA is single-stranded with short double-stranded regions due to appearance of inverted repeats **(b)**.

Visualization of RNA demonstrated that in interphase, transcripts of PO41 repeat appear only in the nuclei, but not cytoplasm, indicating that PO41 RNA probably belongs to nuclear retained ncRNA family. In nuclei wherein PO41 repeat transcription had just begun, one or two major foci of PO41 repeat transcripts presumably represent nascent RNAs. This interpretation is supported by the fact that major PO41 RNA foci can often be associated with the sites of high concentration of the elongating form of RNA polymerase II. In nuclei wherein ongoing transcription had happen, major foci and dispersed PO41 repeat transcripts represent nascent (Figure 8a) and released (Figure 8b) PO41 RNAs correspondingly. Thus newly synthesized PO41 RNA spreads to the nucleoplasm and redistribute in the interchromatin space.

In contrast to lampbrush chromosomes, where multi-loci, widespread transcription of PO41 repeat takes place [10], in somatic and transformed cells we detected mainly one major focus of PO41 repeat transcripts most likely representing one individual transcription unit. The difference can be explained by the presence of a stronger promoter and much lower concentration of specific transcription factors required to initiate transcription of the main transcribed array of PO41 repeat in mitotic cells. Moreover, it seems that both strands of PO41 repeat are transcribed from one locus in a genome due to appearance of inverted copies within PO41 repeat arrays. Indeed, presence of short tracks of inverted PO41 repeats with a transcription in one unit was explicitly shown on lampbrush chromosomes [10].

Notably, both nascent and released PO41 RNAs are predominantly single-stranded, that was demonstrated by the complete transcripts elimination in preparations treated before DNA/RNA FISH with RNase A, which cleaves single-stranded RNAs [35], and in preparations treated after DNA/RNA FISH with RNase H, which cleaves RNA in RNA/DNA hybrids [36]. At the same time, sensitivity to RNase III, which digests double-stranded RNA [37], suggest that released single-stranded PO41 RNA can partly shape double-stranded structures such as hairpin or supercoiled RNA (Figure 8b). Formation of double-stranded structures in PO41 RNAs can be due to appearance of inverted tracks of PO41 repeat within one long transcript that was shown previously [10]. Interestingly, RNase H applied before DNA/RNA FISH cleaved only released PO41 repeat transcripts but not nascent PO41 RNA in the major foci. Thus our observations suggest that PO41 repeat transcripts are probably able to anneal with the relaxed DNA template and to form a duplex similar to RNA transcripts from GAA∙TTC repeat [38].

### To the question of PO41 RNA processing

Subnuclear domains accumulating PO41 RNA do not colocalize with CBs and histone locus bodies in chicken interphase nuclei. Additionally, PO41 repeat transcripts do not recruit hnRNP protein K and do not form nuclear domains similar to nSBs at least in normal conditions. The similar lack of the colocalization of RNA derived from subtelomeric repeat with known nuclear subcompartments in *P. falciparum* was reported [22]. Contrariwise, in human cells, stress induced satellite DNA transcripts are responsible for formation of nSBs, which accumulate hnRNP K [32,33]. Furthermore, RNP matrix of lampbrush chromosomal loops containing C-rich PO41 RNA is enriched with hnRNP K [10].

We also revealed that in cultured cells TMG-capped snRNAs are present in the major PO41 RNA foci although with no specific accumulation. Similarly, in HeLa cells satellite III transcripts localize in a zone with snRNPs without higher concentration forming a complex with snRNPs as shown in immunoprecipitation experiments [39]. At the same time on lampbrush chromosomes of chicken and Japanese quail, allowing cytological observation of co-transcriptional stages of RNA processing, complete failure of snRNAs interaction with PO41 repeat transcripts within RNP-matrix of lateral loops was shown [10]. These data have further implications indicating that processing of PO41 RNAs in somatic cells can be different from processing of transcripts of the same repeat in lampbrush-stage oocytes in terms of co-transcriptional U snRNA-dependent splicing and hnRNP-dependent packaging.

### The behavior of the PO41 RNA during mitosis

To determine the fate of PO41 RNAs, and in particular, to check if transcripts remain associated with chromosomes through mitosis or are disrupted and then resynthesized during interphase, we analyzed the redistribution of PO41 repeat transcripts in cell division and compared it with the redistribution of the other ncRNAs.

In prophase PO41 repeat transcripts are located in the nuclei, while in metaphase are spread between condensed chromosomes and are accessible to cytoplasmic factors. Analogous distribution was shown for GRC-RNAs, C_0_T-1 RNA and X-inactive specific transcript (XIST) RNA, which in human somatic cells were detected as dispersed foci between chromosomes in the cytoplasm at metaphase [35,40,41]. By comparison, *Xist* RNA of rodent mammals remains associated with Xi up to metaphase [42].

Importantly, in anaphase transcripts of PO41 repeat are first oriented at the cleavage plane and then are divided between daughter cells without association with chromosomes. Next, in telophase, more distinct compact clusters of PO41 repeat RNAs, being sensitive to RNase A, RNase H and RNase III treatments, surround terminal regions of chromosomes. According to the data illustrated in the literature, in anaphase the similar pattern of distribution of noncoding transcripts between the cleavage plane was demonstrated for XIST/*Xist* RNA and GRC-RNAs [35,40,42]. However, then transcripts were completely removed and resynthesized again in interphase, only few transcripts foci continue to be detected in the cytoplasm, in the midbodies [35,40]. Preservation of apparently long-lived PO41 RNAs until telophase and their potential to form clusters around chromosome termini may be associated with important functions of subtelomere repeat transcripts at telophase stage. Strikingly, the pattern of distribution of PO41 RNA in anaphase and telophase corresponds to the distribution of mRNA for several protein-coding genes in *Drosophila* [43]*.* For example, mRNA of the *Ste12DOR* gene remains associated with telomeric regions at anaphase. Authors assumed that mRNAs of the *Ste12DOR* gene may function in the “repeat-associated small interfering RNA” (rasiRNA) pathway [43].

## Conclusion

Transcription of a subtelomeric tandem repeat in avian somatic tissues is reported here for the first time. Subtelomere repeat transcripts could influence the telomeric repeat containing RNA (TERRA) synthesis [44]. From the other hand, transcription of both strands of PO41 repeat may result in long-lived ncRNA, which can form RNA duplexes and can enter the cytoplasm during the cell cycle. Whether transcripts of subtelomeric repeat can be involved in the co-transcriptional silencing or mechanisms similar to X-chromosome inactivation remains to be elucidated. The epigenetic status of PO41 repeat arrays in mitotic cells has not been addressed so far. Nevertheless, the presence of constitutive heterochromatic blocks at the subtelomere regions of chicken and Japanese quail chromosomes at least on macrochromosomes 1 and 2 was demonstrated earlier [45,46]. These data and the existence of Dicer-depended pathway of heterochromatin formation in birds [47] provide additional arguments in favor of the potential PO41 RNA participation in RNA-dependent process of heterochromatin formation at subtelomeric regions of chromosomes.

## Methods

### Animals and cell culture

Chicken (*G. gallus domesticus*) and Japanese quail (*C. coturnix japonica*) egg-laying females were taken from poultry farm of the Leningrad region (Russia). All institutional and national guidelines for the care and use of laboratory and farm animals were followed. Six tissues, namely telencephalon, cerebellum, oviduct, small and large intenstine and skeletal muscle, were collected on ice, cut into pieces, pre-fixed in 4% paraformaldehyde in phosphate-buffered saline (PFA/PBS) (1.47 mM KH_2_PO_4_; 4.29 mM Na_2_HPO_4_x7H_2_O; 137 mM NaCl; 2.68 mM KCl) for 4 hours, washed three times for 15 min each in PBS and stored no longer than three weeks at +4°C in PBS with 0.02% sodium azide before use. Control fresh tissue samples were treated with RNase A (50 μg/ml, ThermoScientific) or RiboShredder RNase Blend (50 u/ml, Epicenter Biotechnologies) for 120 min, washed three times for 15 min each in 2×SSC (0.3 M NaCl, 0.03M sodium citrate) and fixed as described above.

MDCC-MSB1 cell line (Marek disease chicken cells, MSB1 – cell line derived from splenic tumors) was provided by the “Bank of cell cultures” of the Institute of Cytology RAS (Russia). Cells were cultured in MEM medium supplemented with 10% FCS and 1% nonessential amino acids. Exponentially growing cells were pelleted at 1000 × g, washed in PBS, and then fixed in 2% PFA in PBS for 15 min. Cells were washed for 5 min in PBS and stored at +4°C in PBS with 0.05% sodium azide. Suspension of MDCC-MSB1 cells was pipetted onto the slides, fixed in 1% PFA in PBS for 10 min in humid chamber, and washed three times for 5 min each in PBS.

### Tissue cryosectioning

Freshly fixed pieces of tissues were mounted into Jung Tissue Medium (Leica Microsystems, Germany) and sectioned by cryostat CM1850UV (Leica Microsystems, Germany). The cryostat chamber temperature varied from –18°C to –23°C. 20 μm (for telencephalon, cerebellum, small and large intestine, muscles) and 40-50 μm (for oviduct) cryosections were mounted on 3-aminopropyltriethoxysilane coated slides and additionally fixed in 2% PFA in PBS for 10 min, then washed three times for 5 min each in PBS and immediately used for FISH.

### Fluorescent *in situ* hybridization

Cy3-labeled DNA oligonucleotide probe for G-rich transcripts of PO41 repeat and Cy5- or biotin-labeled DNA oligonucleotide probes for C-rich transcripts of PO41 repeat (PO41neg and PO41pos respectively) [10,27] and biotin-labeled DNA oligonucleotide probe for U7 snRNA [31] were used for FISH. RNA-transcripts were detected by FISH according to DNA/RNA hybridization protocol. FISH on cryosections allows to investigate cell type-specific transcription in different tissues [48]. Cryosections and MDCC-MSB1 cells were permeabilized with 0.5% Triton X100 for 30 min and 5 min correspondingly, washed once in PBS, and dehydrated in ethanol. FISH according to DNA/DNA hybridization protocol was used as a positive control of labeled probes and detection system. For DNA/DNA FISH preparations were denatured at 82°C for 5 min. Preparations were hybridized with probes (5 ng/μl in hybridization buffer: 40% formamide, 2 × SSC, 10% dextran sulfate, 50-fold excess of salmon sperm DNA or 1 μg/μl of tRNA in the case of multicolor FISH with PO41neg and U7 snRNA probes) overnight at room temperature (RT). Slides were washed once in 2 × SSC and twice in 4 × SSC at 37°C. In the case of biotin-labeled probes, slides were incubated in blocking solution (1% BSA, 4 × SSC with 0.02% Tween) for 40 min at 37°C; then biotin was detected with avidin-Cy3 (1:300, Jackson Immuno Research Laboratories) or avidin-Alexa 488 (Molecular Probes Inc.). The signal was amplified with anti-avidin-biotin (1:200, Vectorlabs) followed by second incubation with the avidin-Cy3 or avidin-Alexa 488. All preparations were dehydrated, air-dried and mounted in antifade solution containing 1 μg/ml 4′,6-Diamidino-2-phenylindole (DAPI).

Autofluorescence of tissue cryosections in channels for both fluorochromes (Cy5 and Cy3) used was examined after all pre-hybridization treatments, dehydratation and mounting in antifade solution containing DAPI. Hybridization with the probes and post-hybridization washes were omitted.

To characterize transcripts structure, specimens were treated with RNases having different substrate specificity: RiboShredder RNase Blend (50 u/ml, Epicenter Biotechnologies), RNase A (50 μg/ml, ThermoScientific), RNase H (100 u/ml, Epicenter Biotechnologies), RNase III (100 u/ml, Epicenter Biotechnologies) before or after hybridization, during 60 min for cells and 120 min for cryosections at 37°C. After FISH, slides were washed as described above and mounted in antifade solution containing 1 μg/ml DAPI.

FISH on 3D preserved tissue fragments was performed in accordance with the protocols of DNA/RNA and DNA/DNA *in situ* hybridization. Tissue fragments were fixed in 4% PFA in PBS and permeabilized with 0.25% Triton X-100 (ICN Biomedicals). Samples for negative control were treated with RNase A (100 μg/ml, ThermoScientific). After a series of washes fixed tissues were incubated for 24 h in prehybridization buffer (2 × SSC, 40% formamide) at RT. Preparations for DNA/DNA hybridization were denatured in buffer (1 × SSC, 70% formamide) for 20 min at 75°C. Tissue fragments were then incubated in hybridization buffer (2 × SSC, 35% formamide, 10% dextran sulfate, 50-fold excess of salmon sperm DNA) with 10 ng/μl fluorochrome-labeled oligonucleotide probe for 24 h at RT. Samples were washed in 4 × SSC at 37°C for 40 min. Preparations were mounted in chambers, containing PBS and DAPI (1 μg/ml), and analyzed by confocal laser scanning microscopy.

### Immunofluorescent staining

For immunofluorescent staining prior to FISH, MDCC-MSB1 cells were treated with 0.5% blocking reagent (BSA) in PBS for 1 h, incubated for 1 h with primary antibodies (Abs) diluted in 0.5% BSA in PBS, washed thrice in PBS, incubated for 1 h with the appropriate secondary Abs diluted in 0.5% BSA in PBS and washed similarly, dehydratated and air dried. FISH with oligonucleotide probes according to DNA/RNA hybridization protocol after immunofluorescent staining was performed as described above.

The following primary Abs were used: rabbit pAb H-300 against coilin (Santa Cruz Biotechnology), mouse mAb against 2,2,7-trimethylguanosine cap (Santa Cruz Biotechnology), mouse mAb 3C2 against heterogeneous nuclear ribonucleoprotein (hnRNP) K/J [49], mouse mAb H14 against phosphorylated C-terminal domain of RNA polymerase II (Abcam). The following secondary Abs were used: Cy3-conjugated goat anti-rabbit IgG (Amersham Life Science), Alexa-488-conjugated goat anti-mouse IgG (Jackson ImmunoResearch Laboratories).

### Confocal laser scanning microscopy and image processing

Fluorescent signals were analyzed using confocal laser scanning microscope Leica TCS SP5 (Leica-Microsystems, Germany), equipped with 40×, 63× and 100× oil objectives. Images were taken at RT with a resolution 1024 × 1024 pixels; each signal was acquired sequentially then merged using LAS AF Software (Leica-Microsystems, Germany). The value of the PMT voltage (“*Gain*” parameter) during image acquisition after DNA/DNA FISH was lower than after DNA/RNA FISH. Merged images, sequential and rotation videos were created by the LAS AF Software. The diameter of nuclear foci with the hybridization signals was measured using the LAS AF Software on the confocal section with the largest size of foci occupied by the fluorochrome (N = 20 for each group). To create 3D reconstructions of MDCC-MSB1 cells, isosurfaces at the outline of fluorescent signals above the background level were constructed using the Imaris Software (Bitplane, Scientific Software; version 7.0.0).

## Additional files

Additional file 1: Figure S1. Comparative analysis of the localization of PO41 RNA major foci and components of RNA transcription and processing in interphase nucleus of chicken MDCC-MSB1 cells. **Figure S2.** Nuclear PO41 RNA foci do not co-localize with Cajal bodies and histone locus bodies in MDCC-MSB1 cells. **Figure S3.** PO41 repeat is transcribed in chicken oviduct cells. **Figure S4.** PO41 repeat is transcribed in chicken cerebellum cells. **Figure S5.** PO41 repeat is transcribed in chicken telencephalon cells. **Figure S6.** C-rich PO41 repeat transcripts in chicken telencephalon cells are sensitive to RNases treatments. **Figure S7.** C-rich PO41 repeat transcripts in chicken small intestine cells are sensitive to RNases treatments. Additional file 2: Table S1. Results of fluorescent *in situ* hybridization with PO41pos and PO41neg probes before or after treatment of MDCC-MSB1 interphase cells with different RNases. **Table S2.** Results of fluorescent *in situ* hybridization with PO41pos and PO41neg probes after treatment of dividing MDCC-MSB1 cells with different RNases. **Table S3.** Results of fluorescent *in situ* hybridization with PO41pos and PO41neg probes on cryosections of chicken somatic tissues. Additional file 3:
**Video 1.** C-rich transcripts of PO41 repeat (*green*) in chicken (*G. g. domesticus*) sceletal muscle cells. Muscle cryosection after RNA FISH with PO41pos probe analyzed with laser scanning confocal microscope. DNA was counterstained with DAPI (*blue*). Movie, showing sequential optical sections of chicken sceletal muscle, was constructed with LAS AF software (Leica-Microsystems CMS GmbH). Additional file 4:
**Video 2.** G-rich transcripts of PO41 repeat (red) in chicken (*G. g. domesticus*) cerebellum cells. Tissue fragment after 3D RNA FISH with PO41neg probe analyzed with laser scanning confocal microscope. DNA was counterstained with DAPI (blue). Movie, showing sequential optical sections of chicken cerebellum, was constructed with LAS AF software (Leica-Microsystems CMS GmbH).

## Abbreviations

Abs: Antibodies
CBs: Cajal bodies
DAPI: 4′,6-Diamidino-2-phenylindole
FISH: Fluorescence *in situ* hybridization
ImmunoFISH: FISH after immunofluorescent staining
hnRNPs: Heterogeneous nuclear ribonucleoproteins
LBCs: Lampbrush chromosomes
nSBs: Nuclear stress bodies
ncRNA: Non-coding RNA
PBS: Phosphate-buffered saline
PFA: Paraformaldehyde
PO41 repeat: Pattern of 41 bp repeat
RT: Room temperature
snRNA: Small nuclear RNA
TMG-capped snRNAs: Trimethylguanosine-capped small nuclear RNAs
