# Supplementary material for: Non-coding RNA derived from a conservative subtelomeric tandem repeat in chicken and Japanese quail somatic cells
Source: Mol Cytogenet. 2014 Dec 23;7:102. doi: 10.1186/s13039-014-0102-7 (PMC4301066; doi:10.1186/s13039-014-0102-7)
Supplement: Additional file 1: Figure S1. — Comparative analysis of the localization of PO41 RNA major foci and components of RNA transcription and processing in interphase nucleus of chicken MDCC-MSB1 cells. Figure S2. Nuclear PO41 RNA foci do not co-localize with Cajal bodies and histone locus bodies in MDCC-MSB1 cells. Figure S3. PO41 repeat is transcribed in chicken oviduct cells. Figure S4. PO41 repeat is transcribed in chicken cerebellum cells. Figure S5. PO41 repeat is transcribed in chicken telencephalon cells. Figure S6. C-rich PO41 repeat transcripts in chicken telencephalon cells are sensitive to RNases treatments. Figure S7. C-rich PO41 repeat transcripts in chicken small intestine cells are sensitive to RNases treatments. [file 13039_2014_102_MOESM1_ESM.pdf]

Additional file 1 as PDF

for the article "**Non-coding RNA derived from a conservative subtelomeric tandem repeat in chicken and Japanese quail somatic cells**", Molecular Cytogenetics, Irina Trofimova, Darya Popova, Elena Vasilevskaya, Alla Krasikova*, Saint-Petersburg State University, alla.krasikova@gmail.com

Figure S1. Comparative analysis of the localization of PO41 RNA major foci and components of RNA transcription and processing in interphase nucleus of chicken MDCC-MSB1 cells.

Combined DNA/RNA FISH with PO41neg probe and immunostaining with antibodies against elongating form of RNA polymerase II (a), protein K/J of hnRNPs (b), TMG-capped snRNAs (c).

G-rich PO41 repeat transcripts are shown in *red*, RNA-polymerase II, protein K and snRNAs – in *green*.

(a-a") Nuclear PO41 RNA foci do not exhibit specific accumulation of TMG-capped snRNAs.

(b-b") Nuclear PO41 RNA foci do not co-localize with foci accumulating protein K/J of hnRNPs.

(c-c") Nuclear PO41 RNA foci co-localize with foci accumulating RNA-polymerase II.

(a"-c") Fluorescence intensity profiles for PO41neg FISH signals and signals after immunostaining with antibodies against RNA polymerase II (a"), protein K (b") and snRNAs (c") along the line through the major nuclear PO41 RNA foci (a', b', c').

DNA was counterstained with DAPI. Scale bars: 2 µm.

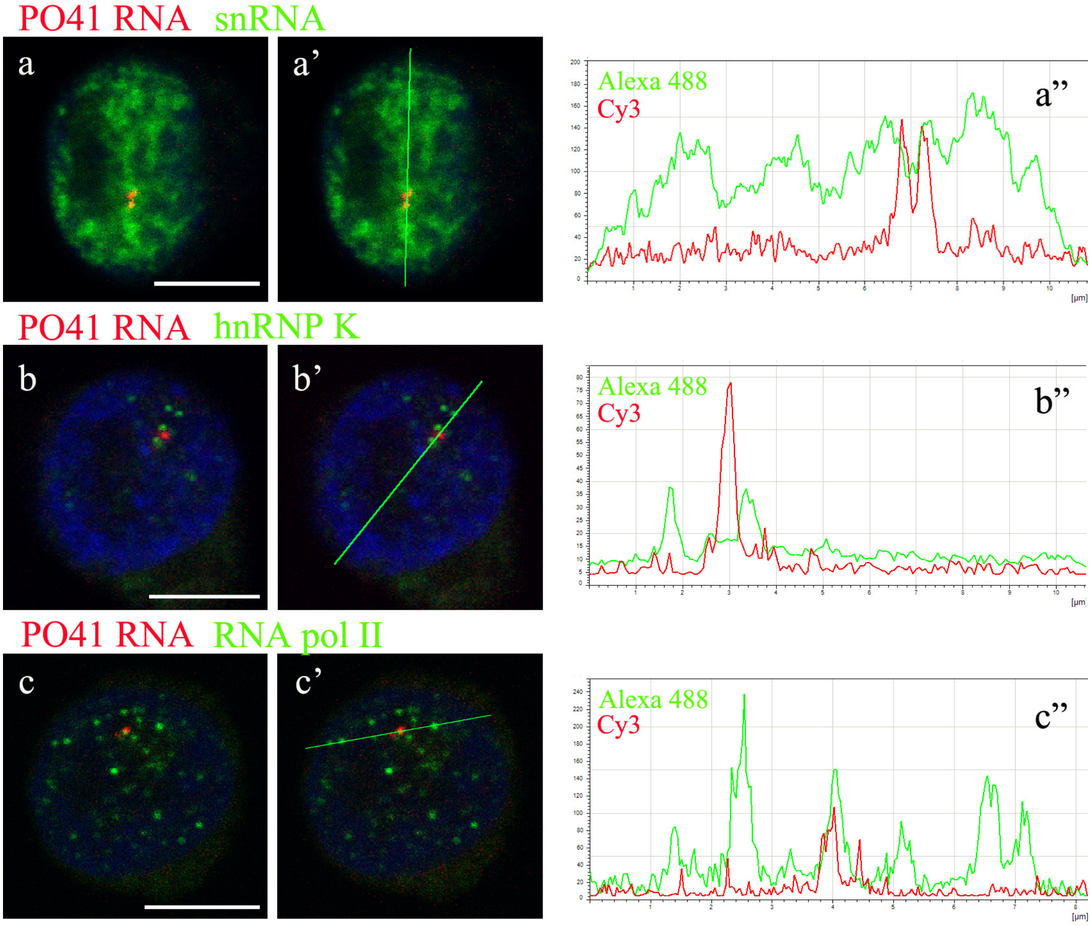
PO41 RNA snRNA
a
a'
Alexa 488
Cy3
a''
PO41 RNA hnRNP K
b
b'
Alexa 488
Cy3
b''
PO41 RNA RNA pol II
c
c'
Alexa 488
Cy3
c''
[μm]

Figure S2. Nuclear PO41 RNA foci do not co-localize with Cajal bodies and histone locus bodies in MDCC-MSB1 cells.

(a) Combined DNA/RNA FISH with PO41neg probe (*red*) and immunostaining with antibodies against coilin (*green*). Major PO41 RNA focus and Cajal bodies (arrows) do not co-localize in MDCC-MSB1 cell nuclei.

(b) DNA/RNA FISH with PO41neg probe (*red*) and U7 snRNA antisense probe (*green*). Major PO41 RNA focus and histone locus body (arrow) do not co-localize in MDCC-MSB1 cell nuclei.

DNA was counterstained with DAPI. Scale bars: 2 µm (a); 5 µm (b).

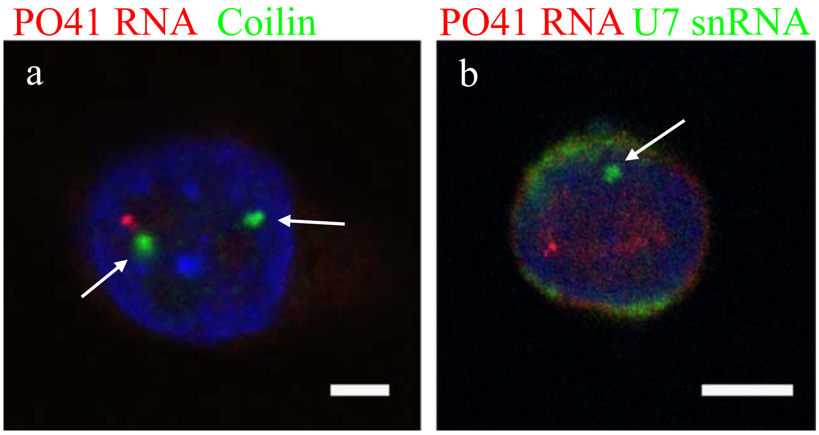

PO41 RNA Coilin
PO41 RNA U7 snRNA
a
b

Figure S3. PO41 repeat is transcribed in chicken oviduct cells.

FISH with PO41pos (*green*, upper row) and PO41neg (*red*, bottom row) probes on oviduct cryosections.

(a, a’) DNA/RNA hybridization revealed transcripts from both strands of PO41 repeat in all cell layers of chicken oviduct.

(b, b’) DNA/DNA hybridization (positive control) revealed clusters of PO41 repeat in all cell nuclei.

(c, c’) RNase A treatment before DNA/RNA hybridization (negative control) removed all hybridization signals.

Nuclei were counterstained with DAPI. Scale bars: 30 μm (a-c, b’, c’); 40 μm (a’).

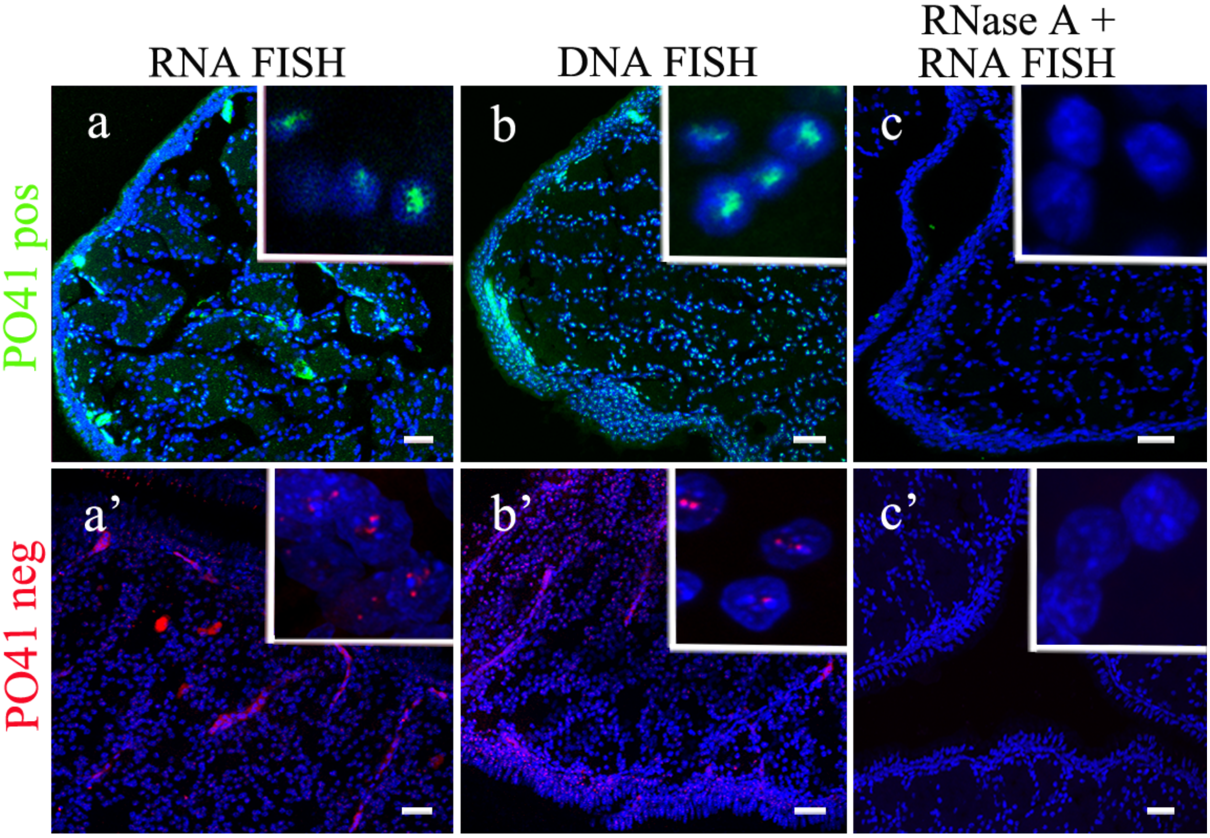
RNA FISH
DNA FISH
RNase A +
RNA FISH
PO41 pos
PO41 neg
a
b
c
a’
b’
c’

Figure S4. PO41 repeat is transcribed in chicken cerebellum cells.

FISH with PO41pos (*green*, upper row) and PO41neg (*red*, bottom row) probes on cerebellum cryosections.

(a, a') DNA/RNA hybridization revealed transcripts from both strands of PO41repeat in all cell layers of chicken cerebellum.

(b, b') DNA/DNA hybridization (positive control) revealed clusters of PO41 repeat in all cell nuclei.

(c, c') RNase A treatment before DNA/RNA hybridization (negative control) removed hybridization signals from PO41pos probe (c), but not from PO41neg probe (c'), especially in the large nuclei of Purkinje neurons. Arrows indicate nuclei of Purkinje neurons.

(d, d') Corresponding controls for autofluorescence.

Nuclei were counterstained with DAPI. Scale bars: 40 µm (a-d, a', c'-d'); 30 µm (b').

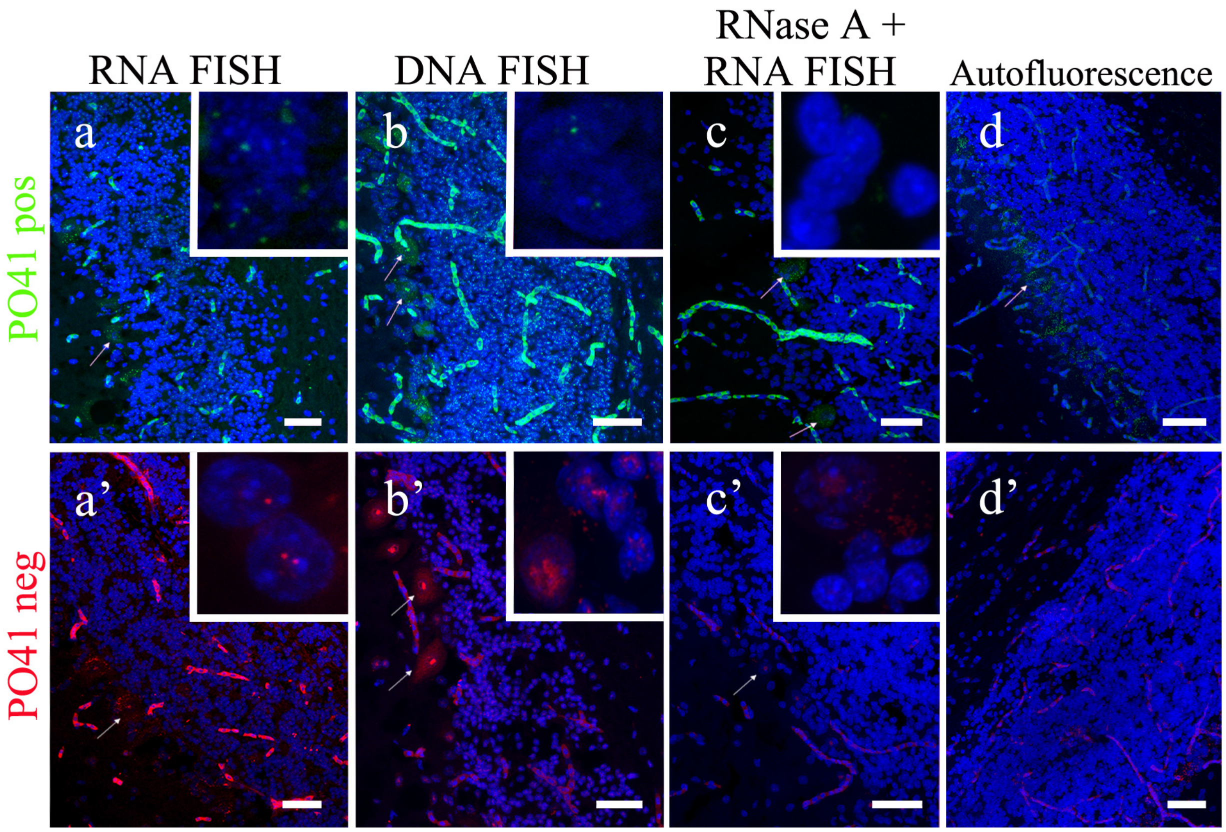

RNA FISH
DNA FISH
RNase A +
RNA FISH
Autofluorescence
PO41 pos
PO41 neg
a
b
c
d
a’
b’
c’
d’

Figure S5. PO41 repeat is transcribed in chicken telencephalon cells.

FISH with PO41pos (*green*, upper row) and PO41neg (*red*, bottom row) probes on telencephalon cryosections.

(a, a’) DNA/RNA hybridization revealed transcripts from both strands of PO41repeat in all cell layers of chicken telencephalon.

(b, b’) DNA/DNA hybridization (positive control) revealed clusters of PO41 repeat in all cell nuclei.

(c, c’) RNase A treatment before DNA/RNA hybridization (negative control) removed hybridization signals from PO41pos probe (c), but not from PO41neg (c’) probe.

Nuclei were counterstained with DAPI. Scale bar: 40 μm.

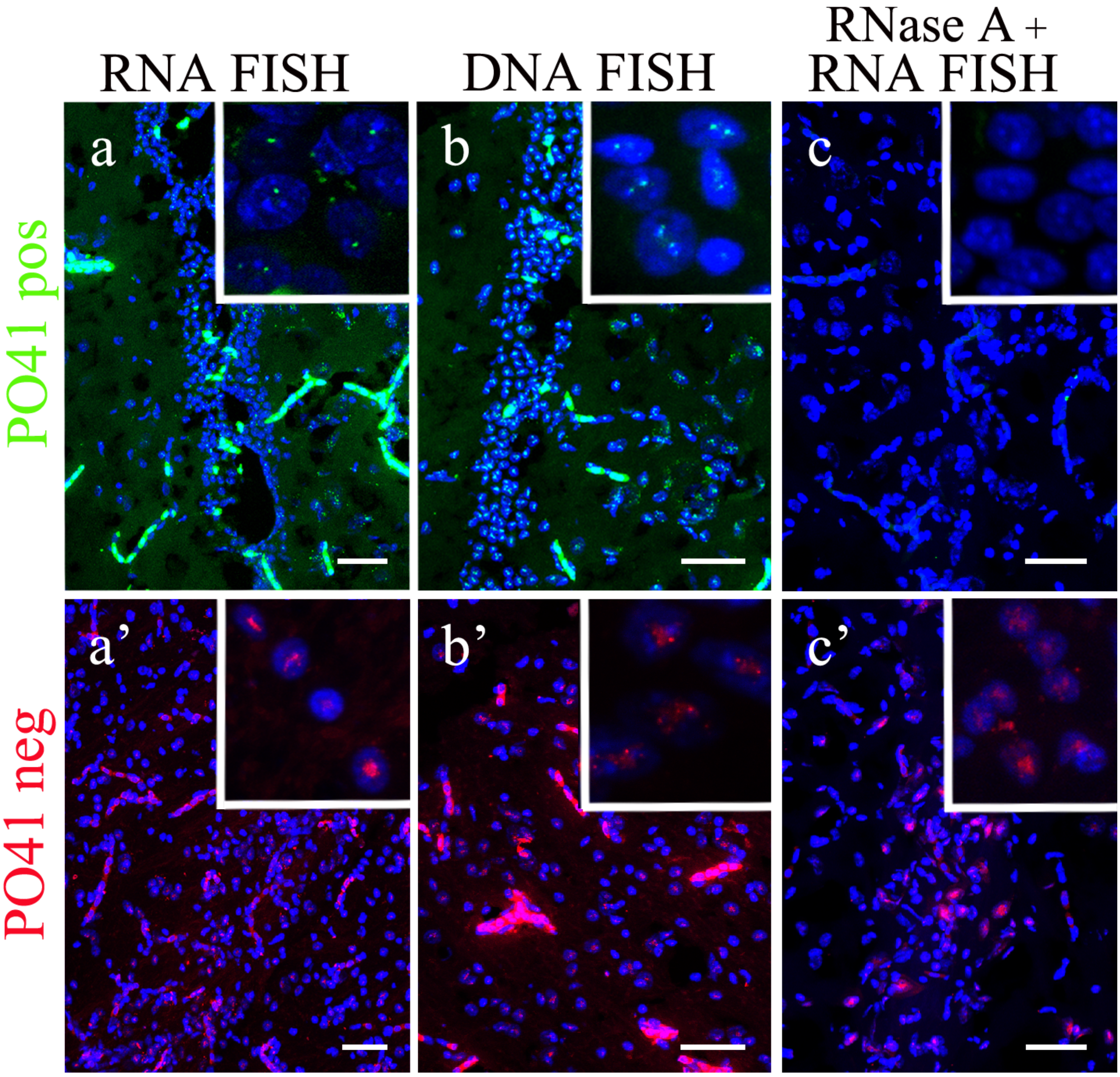
RNA FISH
DNA FISH
RNase A +
RNA FISH
PO41 pos
PO41 neg
a
b
c
a'
b'
c'

Figure S6. C-rich PO41 repeat transcripts in chicken telencephalon cells are sensitive to RNases treatments.

DNA/RNA FISH with PO41pos (*green*, upper row) and PO41neg (*red*, bottom row) probes on telencephalon cryosections. RiboShredder RNase cocktail completely removed signals from PO41pos probe (a), but not from PO41neg probe (a'). RNase H (b, b') and RNase III (c, c') did not remove signals from both probes. Nuclei were counterstained with DAPI. Scale bar: 40 μm.

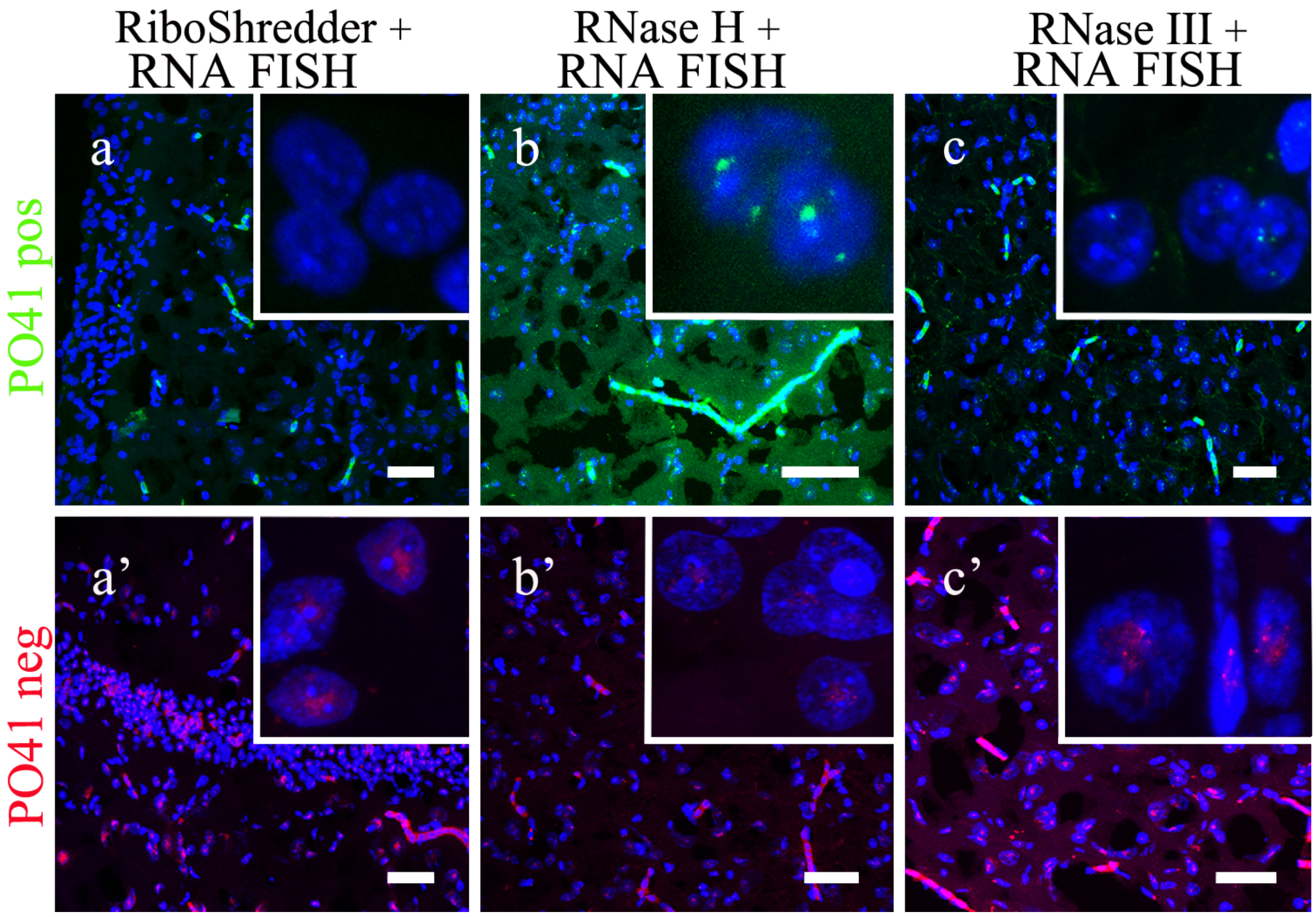

RiboShredder +
RNA FISH
RNase H +
RNA FISH
RNase III +
RNA FISH
PO41 pos
PO41 neg
a
b
c
a’
b’
c’

Figure S7. C-rich PO41 repeat transcripts in chicken small intestine cells are sensitive to RNases treatments.

DNA/RNA FISH with PO41pos (*green*, upper row) and PO41neg (*red*, bottom row) probes on small intestine cryosections. RiboShredder RNase cocktail removed C-rich PO41 repeat transcripts (a), but not labeled cytoplasmic granules (a'). RNase H (b, b') and RNase III (c, c') did not remove signals from PO41pos probe (b, c) and cytoplasmic granules (b', c'). Right-hand images demonstrate enlarged fragments of muscles layer (upper panels) and mucous membrane (bottom panels) of small intestine. Right column (d, d') represents controls for autofluorescence. Nuclei were counterstained with DAPI. Scale bars: 40 μm (a-c, a'); 35 μm (d, b', d'); 25 μm (c').

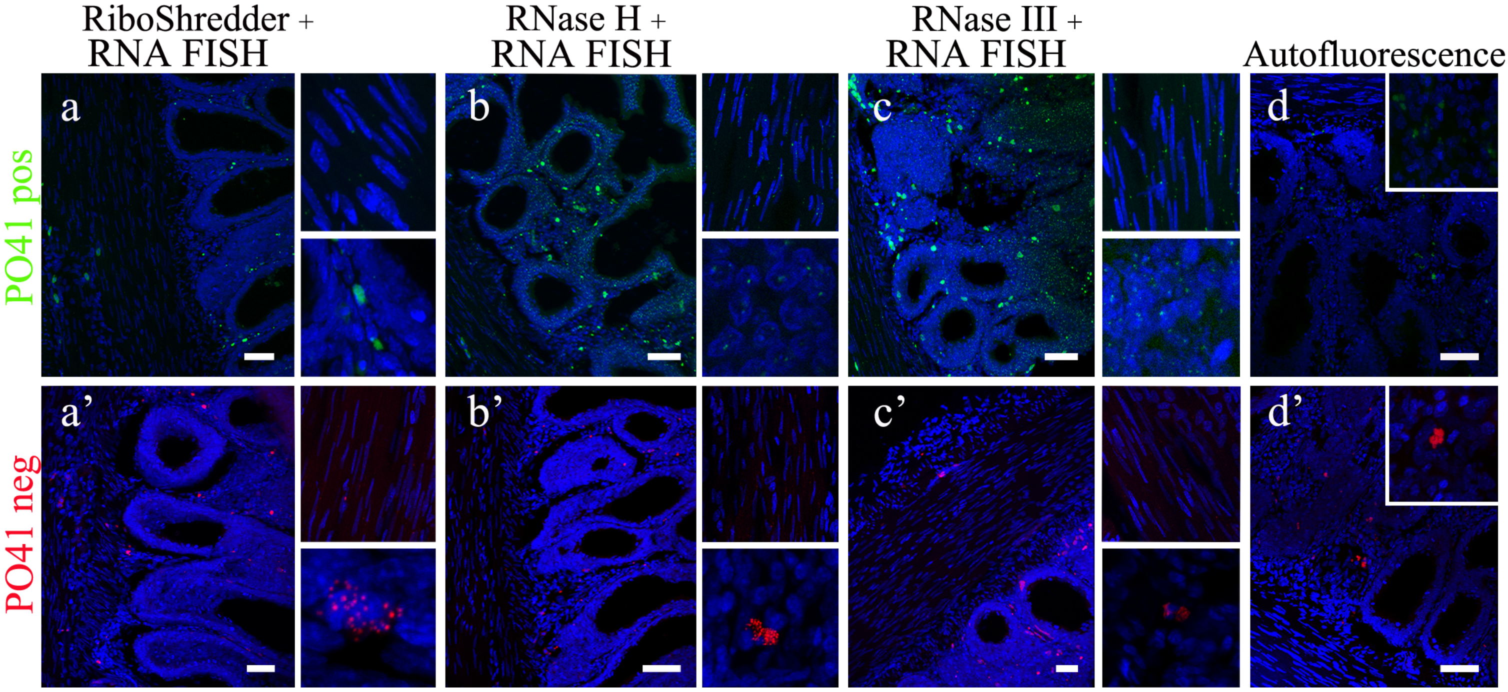
RiboShredder + RNA FISH
RNase H + RNA FISH
RNase III + RNA FISH
Autofluorescence
PO41 pos
PO41 neg
a
b
c
d
a’
b’
c’
d’
