# Supplementary material for: Non-coding RNA derived from a conservative subtelomeric tandem repeat in chicken and Japanese quail somatic cells
Source: Mol Cytogenet. 2014 Dec 23;7:102. doi: 10.1186/s13039-014-0102-7 (PMC4301066; doi:10.1186/s13039-014-0102-7)
Supplement: Additional file 2: Table S1. — Results of fluorescent in situ hybridization with PO41pos and PO41neg probes before or after treatment of MDCC-MSB1 interphase cells with different RNases. Table S2. Results of fluorescent in situ hybridization with PO41pos and PO41neg probes after treatment of dividing MDCC-MSB1 cells with different RNases. Table S3. Results of fluorescent in situ hybridization with PO41pos and PO41neg probes on cryosections of chicken somatic tissues. [file 13039_2014_102_MOESM2_ESM.pdf]

Additional file 2

for the article "Non-coding RNA derived from a conservative subtelomeric tandem repeat in chicken and Japanese quail somatic cells", Molecular Cytogenetics, Irina Trofimova, Darya Popova, Elena Vasilevskaya, Alla Krasikova*, Saint-Petersburg State University, alla.krasikova@gmail.com

Table S1. Results of fluorescent *in situ* hybridization with PO41pos and PO41neg probes before or after treatment of MDCC-MSB1 interphase cells with different RNases.

**Probe**	**Without RNases treatment**	**RNase A**	**RNase III**	**RNase H**	**RNase H***
**PO41pos**	One or two bright foci in the nucleus or signals dispersed in interchromatin space	No signals in the nucleus	One week focus in the nucleus. No dispersed signals in the nucleus	One or multiple foci in the nucleus. No dispersed signals in the nucleus	No signals in the nucleus
**PO41neg**	One or two bright foci in the nucleus or signals dispersed in interchromatin space	No signals in the nucleus	One to three small compact foci. No dispersed signals in the nucleus	One or two bright foci in the nucleus. No dispersed signals in the nucleus	No signals in the nucleus

* In case RNase H treatment performed after RNA-FISH.

Table S2. Results of fluorescent *in situ* hybridization with PO41pos and PO41neg probes after treatment of dividing MDCC-MSB1 cells with different RNases.

**PO41pos probe, C-rich transcripts**				
**RNase**	**Prophase**	**Metaphase**	**Anaphase**	**Telophase**
**RiboShredder RNase blend**	Without signals	Without signals	Without signals	Without signals
**RNase A**	Without signals	Without signals	Without signals	Without signals
**RNase H***	Without signals	Without signals	Without signals	Without signals
**RNase H**	Without signals or with week signals	Predominantly without signals, rarely with one week signal	Without signals	Without signals
**RNase III**	Two signals	Predominantly without signals, rarely with one week signal	Without signals	Without signals
**PO41neg probe, G-rich transcripts**				
**RNase**	**Prophase**	**Metaphase**	**Anaphase**	**Telophase**
**RiboShredder RNase blend**	Without signals	Without signals	Without signals	Without signals
**RNase A**	Without signals	Without signals	Without signals	Without signals
**RNase H***	Without signals	Without signals	Without signals	Without signals
**RNase H**	Without signals	Predominantly without signals, rarely with one week signal	Without signals	Without signals
**RNase III**	Without signals	Predominantly without signals, rarely with one signal	Without signals	Without signals

* In case RNase H treatment performed after RNA-FISH.

Table S3. Results of fluorescent *in situ* hybridization with PO41pos and PO41neg probes on cryosections of chicken somatic tissues.

**Tissue**	**PO41pos probe**			**PO41neg probe**		
	**RNA FISH**	**DNA FISH**	**RNase A + RNA FISH**	**RNA FISH**	**DNA FISH**	**RNase A + RNA FISH**
**Muscles**	One to three foci in nuclei of all cell layers	Multiple foci in nuclei of all cell layers	No signals in the nuclei	One to three foci in nuclei of all cell layers	Multiple foci in nuclei of all cell layers	No signals in the nuclei
**Oviduct**	One or two foci in nuclei of all cell layers	Multiple foci in nuclei of all cell layers	No signals in the nuclei	One or two foci in nuclei of all cell layers	Multiple foci in nuclei of all cell layers	No signals in the nuclei
**Cerebellum**	One or two foci in nuclei of all cell layers	Multiple foci in nuclei of all cell layers	No signals in the nuclei	One or two foci in nuclei of all cell layers	Multiple foci in nuclei of all cell layers	RNase A-stable foci, especially in large nuclei of Purkinje cells
**Telencephalon**	One foci in nuclei of all cell layers	Multiple foci in nuclei of all cell layers	No signals in the nuclei	One foci in nuclei of all cell layers	Multiple foci in nuclei of all cell layers	RNase A-stable foci in nuclei of all cell layers
**Small intestine**	One or two foci in nuclei of mucous membrane	Multiple foci in nuclei of all cell layers	No signals in the nuclei	No signals in the nuclei. Cytoplasmic granules in mucous membrane	Multiple foci in nuclei of all cell layers	Cytoplasmic granules in mucous membrane
**Large intestine**	One foci in nuclei of mucous membrane	Multiple foci in nuclei of all cell layers	No signals in the nuclei	No signals in the nuclei. Cytoplasmic granules in mucous membrane	Multiple foci in nuclei of all cell layers	Cytoplasmic granules in mucous membrane
